# Antimicrobial Peptide Chensinin-1b Suppresses Inflammatory Response Induced by ox-LDL Through Downregulating the Canonical Wnt/β-Catenin Pathway

**DOI:** 10.3390/ijms27083374

**Published:** 2026-04-09

**Authors:** Cen Zhou, Dejing Shang

**Affiliations:** 1 School of Life Science, Liaoning Normal University, Dalian 116081, China; 13054897424@163.com; 2 Liaoning Provincial Key Laboratory of Biotechnology and Drug Discovery, Liaoning Normal University, Dalian 116081, China

**Keywords:** antimicrobial peptide, atherosclerosis, Wnt/β-catenin signaling pathway, inflammation, lipid accumulation

## Abstract

Inflammation occurs throughout the entire process of atherosclerosis. So, reducing the inflammatory response remains one of the main therapeutic strategies for atherosclerosis. Chensinin-1b, a derivative of the natural antimicrobial peptide extracted from the skin secretions of the *Rana chensinensis,* has been shown to effectively mitigate the occurrence of inflammatory responses. Here, the anti-inflammatory activity of the peptide by suppressing the canonical Wnt/β-catenin signaling pathway was investigated in ox-LDL-induced AS. The results showed that the classical Wnt/β-catenin signaling pathway was activated in ox-LDL-induced THP-1-derived foam cells. The inhibitor of Wnt reduced the release of pro-inflammatory cytokines by downregulating the NF-κB signaling pathway. Cyclooxygenase-2, a target protein of the canonical Wnt/β-catenin signaling pathway, inhibited the phosphorylation of NF-κB. Chensinin-1b and its analogs effectively downregulated the expression of Wnt signaling proteins by inhibiting the nuclear translocation of the key pathway protein β-catenin, resulting in a decrease in COX-2 expression and simultaneously reducing the release of pro-inflammatory cytokines. In summary, our study suggests the potential of chensinin-1b and its analogs as therapeutic agents for AS.

## 1. Introduction

Atherosclerosis (AS) is a clinical inflammatory disease in which an inflammatory response occurs throughout the entire process of its occurrence, development, and plaque rupture [1]. After vascular endothelial damage, low-density lipoprotein cholesterol (LDL-C) enters the vessel wall and is oxidized, triggering the activation of the immune system and initiating a continuous inflammatory cascade reaction [2]. This process not only promotes plaque formation but also directly affects its stability. A development of anti-atherosclerotic drugs targeting inflammation is reshaping the prevention and treatment landscape of cardiovascular diseases and showing great potential.

The canonical Wnt/β-catenin signaling pathway is highly conserved in a cascade that regulates cell proliferation, differentiation, apoptosis, and migration [3,4], and its aberrant activation has been implicated in a variety of diseases, including malignancy, metabolic disease, heart failure, and atherosclerosis [5,6,7,8,9,10,11,12]. Studies have found that the canonical Wnt/β-catenin pathway is involved in many different stages of the atherosclerosis (AS) disease process that impact vascular inflammation, oxidative stress, endothelial dysfunction and lipid deposition [12,13]. Secreted frizzled-related protein 4 (SFRP4), as a Wnt inhibitor, reduced lipid accumulation in the aortic root of high-fat diet-fed *ApoE^−/−^* mice by downregulating mRNA expression of pro-inflammatory cytokines such as IL-1, IL-6, IL-17, and TNF-α [14]. Sclerostin is a secreted cysteine-knot protein that interferes with the LRP5/6-Fz complex formation and inhibits Wnt pathway activation [15,16]. Overexpression of sclerostin reduces the inflammatory markers such as TNF-α, IL-6, and IL-10 in plasma, and concentrations of monocyte chemoattractant protein-1 in sclerostin overexpressing mice [17]. Although the canonical Wnt/β-catenin signaling pathway is not typically regarded as a classical inflammatory pathway, accumulating evidence suggests that it plays a positive role in promoting inflammatory processes through crosstalk with multiple classical pro-inflammatory signaling pathways. Cyclooxygenase-2 (COX-2) has been confirmed as a downstream protein of the canonical Wnt/β-catenin signaling pathway [18,19,20,21]. Relevant studies found that the upregulation of COX-2 increased expression of NF-κB-p65 and IKKα in malignant colorectal epithelial cells, supporting that NF-κB activation is induced by COX-2 [22,23]. These studies suggested that COX-2 may be a key protein that links the canonical Wnt/β-catenin signaling pathway with the NF-κB signaling pathway, promoting the release of various inflammatory factors and inducing a robust inflammatory response.

Identifying small-molecule modulators of Wnt signaling looks to be a promising strategy for reducing inflammation in AS. Antimicrobial peptides chensinin-1b and its analog 3–13 and W3R6 are modified peptides obtained by amino acid substitution based on chensinin-1, a new natural antimicrobial peptide isolated from the skin secretion of Chinese forest frog [24]. Chensinin-1b has seven net positive charges, which is the same as chensinin-1, but has improved hydrophobicity compared to the previous one. The results of secondary structure determination showed that chensinin-1b formed an α-helix secondary structure between positions 3 and 13 at the -NH_2_ terminus. W3R6 was synthesized by intercepting the amino acids at positions 3–13 of chensinin-1b and replacing the histidine (H) at positions 4 and 10 with arginine (R). The net positive charge number of W3R6 is six, which is one less than that of chensinin-1b, indicating that its hydrophobicity has been improved [25,26]. The three antimicrobial peptides exhibited broad antibacterial activities [26,27] and anti-inflammatory activity in ox-LDL-induced AS. Importantly, they could inhibit the canonical Wnt/β-catenin signaling pathway. Here, the anti-inflammatory activity of chensinin-1b and its analogs and signal pathway was investigated by regulating the canonical Wnt/β-catenin pathway. We hope to provide a new idea for the development of anti-atherosclerotic drugs.

## 2. Results

### 2.1. Wnt/β-Catenin Signaling Pathway Was Activated in the ox-LDL-Induced Foaming THP-1 Cells

**Figure 1:**
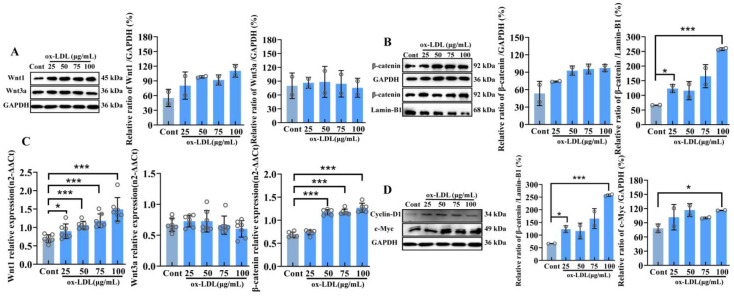
The influence of ox-LDL treatment on the expression of proteins related to the canonical Wnt/β-catenin signaling pathway in PMA-induced human macrophage-like THP-1 cells. (**A**) Western blot analysis of canonical Wnt ligand expression in PMA-differentiated THP-1 macrophages after 24 h treatment with ox-LDL (0–100 μg/mL); (**B**) nuclear/cytoplasmic expression of β-catenin protein; (**C**) qRT-PCR analysis of mRNA expression of Wnt signaling pathway-related proteins; (**D**) downstream protein expression of the Wnt pathway; *: Cont/ox-LDL; *: *p* < 0.05; ***: *p* < 0.001).

PMA-induced human THP-1 macrophages were treated with different concentrations of ox-LDL (0, 25, 50, 75, 100 μg/mL) for 24 h, and the expression of canonical Wnt signal pathway proteins was detected by WB experiment. As shown in Figure 1A, although ox-LDL upregulated the expression of the Wnt ligand Wnt1, exhibiting a concentration-dependent trend, the results were not statistically significant (*p* > 0.05), and it had no effect on the expression of Wnt3a. β-catenin is a key nuclear transcription factor in the canonical Wnt signaling pathway [5,28,29]. After 24 h of treatment with a series of ox-LDL concentrations (0–100 μg/mL), the expression of β-catenin protein in the cytoplasm was increased, but there was no statistically significant difference compared with the control group (*p* > 0.05) (Figure 1B). However, it was found that the expression of β-catenin protein in the nucleus gradually increased with the increase in ox-LDL treatment concentration. An amount of 25 and 100 μg/mL of ox-LDL significantly upregulated the expression of β-catenin protein in the nuclear (*p* < 0.05), and the increase rate was about 110% and 300% of the control group.

The result of RT-qPCR exhibited the similarity with those of WB. As shown in Figure 1C, the relative mRNA expression levels of Wnt1 upregulated with the increase in ox-LDL concentration (*p* < 0.05), but the expression of Wnt3a had not changed (*p* > 0.05). Compared to the control group, 100 μg/mL of ox-LDL upregulated the mRNA expression level of Wnt1 by 110% (*p* < 0.001). After treatment with a high concentration of ox-LDL, it was observed that the relative mRNA expression level of β-catenin also showed a highly significant increasing trend (*p* < 0.001), with the relative mRNA expression level of β-catenin approximately doubled compared to the control group.

Cyclin-D1 and c-Myc are the target proteins of the canonical Wnt signaling pathway [10,30]. Furthermore, the expression of cyclin-D1 and c-Myc was investigated by WB. The results showed that ox-LDL significantly upregulated the expression of cyclin-D1 and c-Myc (Figure 1D). After treatment with the highest concentration (100 μg/mL) of ox-LDL for 24 h, the expression of cyclin-D1 and c-Myc increased by more than 60% (*p* < 0.05). The above results indicated that ox-LDL could activate the canonical Wnt/β-catenin signaling pathway in THP-1 cells undergoing foam cell formation.

### 2.2. Wnt/β-Catenin Signaling Pathway Regulates the Inflammatory Response and Cholesterol Level in the PMA-Induced Human THP-1 Macrophages

#### 2.2.1. Activation of Wnt/β-Catenin Signaling Pathway Promotes the Inflammatory Response and Cholesterol Level in the PMA-Induced Human THP-1 Macrophages

LiCl is an agonist of the Wnt/β-catenin signaling pathway [31,32]. To investigate the effect of the canonical Wnt/β-catenin signaling pathway on the inflammatory response and cholesterol level in the PMA-induced human THP-1 macrophages, LiCl was first used to activate the Wnt/β-catenin signaling pathway, and ox-LDL was used as a positive control.

**Figure 2:**
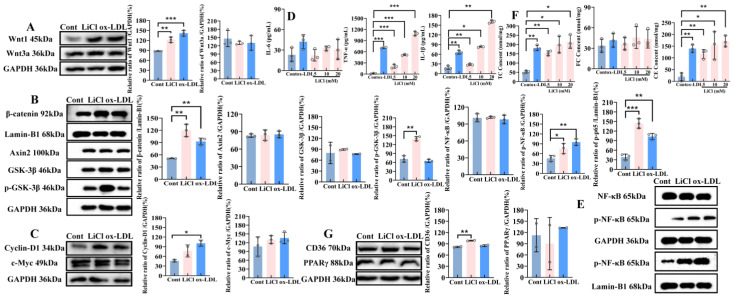
Activation of the Wnt/β-catenin signaling pathway promotes the inflammatory response and cholesterol level in the PMA-induced human THP-1 macrophages. (**A**–**C**) Western blot analysis of canonical Wnt signaling pathway-related protein expression following treatment with 20 mM LiCl and 100 μg/mL ox-LDL; (**D**) inflammatory cytokine release; (**E**) WB analysis of NF-κB protein expression and phosphorylation levels; (**F**) TC, FC, CE; (**G**) expression of lipid metabolism-related proteins; *: Cont/ox-LDL; *: *p* < 0.05; **: *p* < 0.01; ***: *p* < 0.001).

LiCl upregulated the expression of Wnt1 by 35% compared to the control, but was not significantly different from the ox-LDL-treated group (100 μg/mL). Neither LiCl nor ox-LDL treatment had an effect on the Wnt3a expression (*p* > 0.05) (Figure 2A). As a commonly used agonist of the canonical Wnt/β-catenin signaling pathway, LiCl remarkably promoted the nuclear translocation of β-catenin protein (*p* < 0.01), with the nuclear translocation rate approximately doubling that of the control group. Although ox-LDL, like LiCl, also promoted the nuclear translocation of β-catenin, its effect was less pronounced than that of the classical agonist LiCl, only increasing the nuclear translocation rate to approximately 80% of that in the control group; the difference remained highly statistically significant (*p* < 0.01). Axin2 and GSK-3β are constituents of the destructive complexes in the canonical Wnt/β-catenin signaling pathway [33]. The WB result showed that LiCl and ox-LDL do not change the expression of Axin2 and GSK-3β (*p* > 0.05). However, LiCl significantly promoted the phosphorylation of GSK-3β at the Ser 9 site, with the phosphorylation level of GSK-3β increasing to twice as high as that of the control group (*p* < 0.01) (Figure 2B). Moreover, the effect of LiCl on the phosphorylation of GSK-3β (*p* < 0.05) was higher than that of ox-LDL.

The activation of the canonical Wnt/β-catenin signaling pathway by ox-LDL was confirmed by the expression of cyclin-D1. As a downstream protein of this canonical pathway, the expression level of cyclin-D1 increased by 120% compared with the control group (*p* < 0.05) (Figure 2C).

In the ox-LDL-induced foaming macrophages, a large number of pro-inflammatory factors are released by the activation of the NF-κB signaling pathway, and found that 100 μg/mL ox-LDL treatment resulted in a very significant increase in TNF-α and IL-1β release (*p* < 0.01), with an increase rate of about 700% or 275% compared with the control group. After a treatment of different concentrations (5, 10 and 20 mM) of LiCl for 24 h, the release of IL-6 was increased, but not statistically significant (*p* > 0.05). Treatment with LiCl had the greatest effect on TNF-α release, and the increasing rates were about 200%, 500% and 1100% (*p* < 0.001). The release of IL-1β was also significantly increased in response to the stimulation of LiCl (*p* < 0.01), with an increase rate of around 75%, 350%, and 775% compared to the control group (Figure 2D).

Phosphorylation of NF-κB p65, the key protein in the NF-κB inflammatory signaling pathway, is a crucial step for its activation and nuclear translocation, which can promote the sustained activation of inflammatory responses [34,35]. The results showed that neither LiCl nor ox-LDL treatment significantly affected the expression of total NF-κB in the cytoplasm (*p* > 0.05). However, treatment with 20 mM LiCl significantly promoted the increase in NF-κB p65 phosphorylation levels (*p* < 0.05), with the phosphorylation level of p65 increasing to approximately 60% of that in the control group (Figure 2E). Furthermore, LiCl treatment more markedly facilitated the nuclear translocation of phosphorylated p65, with the level of nuclear phosphorylated p65 reaching approximately 275% of that in the control group (*p* < 0.001). This finding explains why treatment with a high concentration of 20 mM LiCl increased the release of the inflammatory cytokine TNF-α to more than 1000 times that of the control group.

To verify the effect of the activation of the canonical Wnt/β-catenin signaling pathway on cholesterol levels in PMA-induced THP-1 macrophages, the cholesterol ester method was used to detect the levels of total cholesterol (TC), free cholesterol (FC), and calculate the changes in cholesterol ester (CE) levels. The results showed that the levels of TC and CE in cells were significantly increased in the positive control group with 100 μg/mL ox-LDL (*p* < 0.01), rising to 2.5 and 8 times that of the control group, but did not significantly affect the expression of FC (Figure 2F). After treatment with different concentrations (5, 10, and 20 mM) of LiCl for 24 h, it was found that the levels of TC and CE were significantly increased in PMA-induced THP-1 macrophages (*p* < 0.05), with the increase rates being approximately 200%, 300%, and 350% compared to the control group. An amount of 20 mM of LiCl exhibited a similar effect on the accumulation of cholesterol levels to the ox-LDL control group. Additionally, Western blot (WB) results indicated that treatment with LiCl and ox-LDL could promote the upregulation of CD36 expression in PMA-induced THP-1 macrophages. However, compared to the control group, a statistically significant increase in CD36 expression (approximately 20%) was only observed with LiCl 20 mM treatment (*p* < 0.01). The expression of PPARγ, a transcription factor of CD36, did not show significant changes after treatment with LiCl and ox-LDL (Figure 2G).

#### 2.2.2. Inhibition of Wnt/β-Catenin Signaling Pathway Reduces the Inflammatory Response and Cholesterol Level in the PMA-Induced Human THP-1 Macrophages

**Figure 3:**
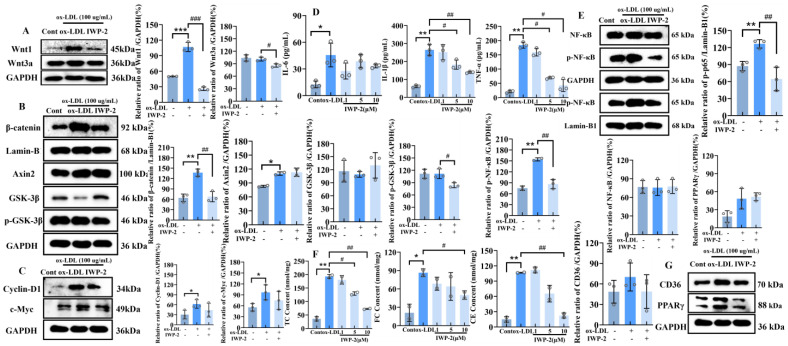
Inhibition of Wnt/β-catenin signaling pathway reduces the inflammatory response and cholesterol level in the PMA-induced human THP-1 macrophages (**A**–**C**) Western blot analysis of canonical Wnt signaling pathway-related protein expression following co-treatment with 100 μg/mL ox-LDL and 10 μM IWP-2; (**D**) inflammatory cytokine release; (**E**) key protein expression of the NF-κB signaling pathway; (**F**) TC, FC, CE; (**G**) expression of lipid metabolism-related proteins; *: Cont/ox-LDL; *: *p* < 0.05; **: *p* < 0.01; ***: *p* < 0.001; #: ox-LDL/IWP-2; #: *p* < 0.05; ##: *p* < 0.01; ###: *p* < 0.001).

IWP-2 is an inhibitor of the canonical Wnt/β-catenin signaling pathway, primarily acting by inhibiting the palmitoylation process of Wnt ligands [36,37]. To explore the effects of the canonical Wnt/β-catenin signaling pathway on the inflammatory response and cholesterol levels in PMA-induced human THP-1 macrophages, IWP-2 was first used to inhibit the Wnt/β-catenin signaling pathway, and the expression changes in key proteins in the canonical Wnt signaling pathway were examined. Incubation with 100 μg/mL of ox-LDL significantly enhanced the expression of Wnt1 (*p* < 0.001), with an increase rate of approximately 120%, but had no significant effect on the expression of Wnt3a. The addition of 10 μM IWP-2 resulted in a marked decrease in the expression of both Wnt1 and Wnt3a ligands, with a more pronounced effect observed on Wnt1 (*p* < 0.001) (Figure 3A). Using Western blotting, it was detected that ox-LDL treatment significantly promoted the nuclear translocation of β-catenin, a key protein in the canonical Wnt/β-catenin signaling pathway (*p* < 0.01). Under these conditions, the nuclear translocation rate of β-catenin approximately doubled that of the control group. However, co-treatment with IWP-2 and ox-LDL significantly inhibited the nuclear translocation of this key protein, bringing its nuclear translocation rate to a level comparable to that of the control group (*p* < 0.01). This indicates that treatment with IWP-2 can effectively inhibit the activation of the canonical Wnt/β-catenin signaling pathway. Furthermore, we examined the effect of IWP-2 treatment on the expression of key components of the β-catenin degradation complex. It was found that treatment with IWP-2 reduced the phosphorylation level of GSK-3β at the Ser9 site by approximately 33%, a statistically significant difference compared to the ox-LDL-only treatment group (*p* < 0.05). However, IWP-2 treatment did not significantly affect the expression of the degradation complex members Axin2 and GSK-3β (*p* > 0.05) (Figure 3B). Additionally, the expression of target proteins Cyclin-D1 and c-Myc of the canonical Wnt/β-catenin signaling pathway decreased after treatment with IWP-2. Compared to the ox-LDL treatment group alone, the expression of both Cyclin-D1 and c-Myc decreased by approximately 25% (Figure 3C). These results indicate that IWP-2 can indeed inhibit the canonical Wnt/β-catenin signaling pathway by suppressing the expression of canonical ligands such as Wnt1, reducing the phosphorylation of GSK-3β, and significantly decreasing the nuclear translocation of the key protein β-catenin.

As shown in Figure 3D, IWP-2 significantly reduced the release of pro-inflammatory factors TNF-α and IL-1β in a concentration-dependent manner. Compared to the ox-LDL group, the release of TNF-α was reduced by 58% and 70% in the PMA-induced human THP-1 macrophages co-treated with 5 and 10 μM of IWP-2 and 100 μg/mL of ox-LDL. Similarly, the treatment with different concentrations of IWP-2 significantly reduced the expression of IL-1β by 4%, 25%, and 40% (*p* < 0.01). However, it was found that co-treatment with different concentrations of IWP-2 did not significantly affect the expression of IL-6, although ox-LDL could significantly promote the release of IL-6 (*p* < 0.05), with an increase rate of 275%. The effects of IWP-2 treatment on the phosphorylation levels of NF-κB protein were also examined. Neither ox-LDL nor IWP-2 treatment significantly affected the expression of the NF-κB p65 (*p* > 0.05). However, ox-LDL treatment significantly promoted the phosphorylation of p65, with the phosphorylation level approximately doubling that of the control group (*p* < 0.01). In contrast, co-treatment with IWP-2 and ox-LDL significantly reversed the ox-LDL-induced increase in p65 phosphorylation, returning the phosphorylation level to a value comparable to that of the control group. This demonstrates that IWP-2 treatment can significantly inhibit the phosphorylation and activation of the NF-κB p65 subunit, a key protein in the pro-inflammatory signaling pathway. Furthermore, we examined the nuclear translocation of the phosphorylated p65 subunit. The results showed that ox-LDL treatment significantly promoted the nuclear translocation of the phosphorylated p65 subunit (*p* < 0.01). Conversely, IWP-2 exerted an inhibitory effect on this nuclear translocation, reducing the nuclear translocation rate of the p65 subunit to approximately half that of the ox-LDL-only treatment group (*p* < 0.01) (Figure 3E). Moreover, the nuclear translocation rate was also lower than that of the control group. Since the phosphorylated p65 subunit exerts its function upon nuclear entry, these findings indicate that the activation of the pro-inflammatory signaling pathway was significantly inhibited.

After co-treating PMA-induced human THP-1 macrophages with ox-LDL and various concentrations of IWP-2 (1, 5, and 10 μM) for 24 h, the total cholesterol (TC) and free cholesterol (FC) contents in the cells were measured using a cholesterol assay kit, and the cholesterol ester (CE) content was calculated. The results showed that the levels of TC, FC, and CE in the cells treated with ox-LDL increased approximately 3-fold (*p* < 0.05) compared to the control group. Following co-treatment with IWP-2, the most pronounced effect was observed at a concentration of 10 μM, which reduced the intracellular levels of TC, FC, and CE by approximately 62.5%, 41%, and 75% (Figure 3F). The expression of key proteins related to lipid metabolism, CD36 and PPARγ, showed no significant changes following treatment with ox-LDL and IWP-2 (Figure 3G).

### 2.3. COX-2 as a Key Downstream Protein of the Canonical Wnt/β-Catenin Signaling Pathway, Can Crosstalk with the NF-κB Pro-Inflammatory Signaling Pathway to Influence the Occurrence of Inflammatory Responses in THP-1 Foam Cells

**Figure 4:**
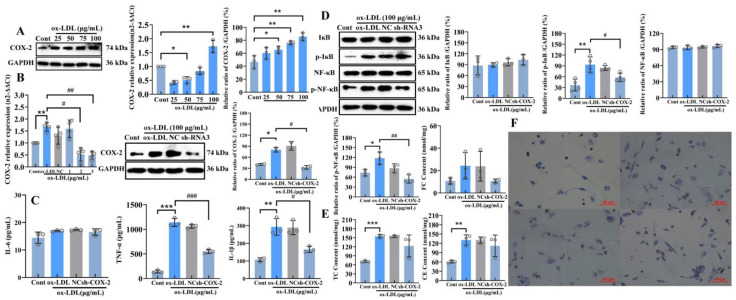
Effects of COX-2 inhibition on inflammation and lipid metabolism. (**A**,**B**) Relative protein and mRNA expression of COX-2; (**C**) inflammatory cytokine release; (**D**) key protein expression of the NF-κB signaling pathway; (**E**) TC, FC, CE; (**F**) Oil Red O staining (Scale bar = 40 µm); *: Cont/ox-LDL; *: *p* < 0.05; **: *p* < 0.01; ***: *p* < 0.001; #: ox-LDL/IWP-2; #: *p* < 0.05; ##: *p* < 0.01; ###: *p* < 0.001).

After treating THP-1-derived macrophages with 25, 50, 75, and 100 μg/mL ox-LDL, it was found that ox-LDL promoted the upregulation of both the mRNA and protein expression levels of COX-2, a key downstream protein of the canonical Wnt/β-catenin signaling pathway, in a dose-dependent manner. The most significant promoting effect was observed with 100 μg/mL ox-LDL treatment (*p* < 0.01), at which point COX-2 expression increased to approximately 80% of that in the control group (Figure 4A).

In molecular mechanism studies, to investigate the crucial role of specific proteins in signal transduction or phenotypic development, it is often necessary to employ a loss-of-function strategy for validation. Therefore, to explore the crosstalk between COX-2, a key downstream protein of the canonical Wnt/β-catenin signaling pathway, and key functional proteins in the NF-κB pathway, we conducted rigorous validation by establishing a scrambled control group and a COX-2 sh-RNA inhibition group. This approach achieved partial knockdown, rather than complete knockout, of specific gene expression in a classical foam cell model of atherosclerosis, thereby constructing a gene function attenuation effect that more closely mimics pathological conditions and providing reliable experimental support for establishing a clear upstream–downstream causal relationship. qRT-PCR was used to verify the knockdown efficiency of three sh-RNAs. The results showed that both sh-RNA2 and sh-RNA3 effectively inhibited the increase in COX-2 expression induced by ox-LDL treatment (*p* < 0.01). Analysis revealed that the knockdown efficiency of sh-RNA3 was higher than that of sh-RNA2, reducing COX-2 mRNA expression to approximately 80% of that in the ox-LDL-treated group (*p* < 0.01); therefore, this sh-RNA was selected for subsequent experiments. WB results showed that ox-LDL treatment significantly promoted COX-2 protein expression, with an increase of approximately 1-fold compared to the control group (*p* < 0.05). In contrast, co-treatment with sh-RNA3 and ox-LDL reduced COX-2 expression to a level similar to that of the control group, indicating that COX-2 expression was effectively inhibited at this point (*p* < 0.05), with the results detailed in Figure 4B.

Following shRNA plasmid transfection-mediated inhibition of COX-2 expression, ELISA was employed to assess inflammatory cytokine release. The results demonstrated that ox-LDL treatment significantly promoted the release of the inflammatory cytokines TNF-α and IL-1β (*p* < 0.01), with the release levels of these two cytokines increasing to approximately 10-fold and 3-fold of those in the control group. However, upon effective inhibition of COX-2 expression, the release of both TNF-α and IL-1β showed a significant decreasing trend (*p* < 0.05), with their levels reduced to approximately half of those observed in the ox-LDL treatment alone group. Notably, neither ox-LDL treatment nor inhibition of COX-2 expression affected the release of the inflammatory cytokine IL-6 (Figure 4C). Subsequently, WB was performed to assess changes in the phosphorylation levels of IκBα, a key rate-limiting protein in the NF-κB signaling pathway, as well as the phosphorylation levels of p65. The results indicated that neither ox-LDL treatment nor COX-2 inhibition significantly affected the total protein expression levels of the NF-κB p65 subunit or IκBα (*p* > 0.05). However, ox-LDL treatment led to a significant increase in IκBα phosphorylation (*p* < 0.01). Following its phosphorylation, IκBα undergoes degradation mediated by E3 ubiquitin ligase, accompanied by the release and subsequent phosphorylation activation of the NF-κB p65 subunit (*p* < 0.05), indicating that the NF-κB signaling pathway was activated under these conditions. Importantly, upon co-treatment with the COX-2 shRNA plasmid and ox-LDL, it was observed that when COX-2 expression was effectively inhibited, the phosphorylation level of IκBα was significantly reduced, with a decrease of approximately 35% compared to the ox-LDL-treated group (*p* < 0.05). Furthermore, the results revealed that inhibiting COX-2 expression had an even more pronounced effect on the phosphorylation of the NF-κB p65 subunit (*p* < 0.01), highly significantly suppressing its phosphorylation to less than half of the level observed in the ox-LDL-treated group (Figure 4D).

Having investigated the molecular mechanism by which COX-2, a key downstream protein of the canonical Wnt/β-catenin signaling pathway, crosstalks with the NF-κB inflammatory signaling pathway to promote inflammatory responses in foam cells, we next sought to explore the impact of this key pathway protein on lipid metabolism in foam cells. To this end, a cholesterol enzymatic assay was performed. The results showed that ox-LDL treatment significantly promoted the accumulation of total cholesterol and cholesterol esters in THP-1 cells, with intracellular cholesterol levels increasing to approximately 1-fold higher than those in the control group (*p* < 0.01). However, upon transfection with the COX-2 shRNA plasmid to inhibit COX-2 expression, although intracellular total cholesterol and cholesterol ester levels were reduced to some extent, the results were not statistically significant (*p* > 0.05) (Figure 4E). This suggests that COX-2, a key downstream protein of the canonical Wnt/β-catenin pathway, may primarily exert its pro-atherosclerotic effects through crosstalk with the NF-κB inflammatory signaling pathway, with a less significant association with intracellular lipid accumulation.

Observation of intracellular red lipid droplet accumulation following Oil Red O staining revealed that after PMA stimulation, THP-1 cells began to adhere to the culture plate surface. The cell bodies spread out, volume increased significantly, and they extended pseudopodia or transformed into various irregular shapes, such as spindle or fusiform forms, exhibiting the typical ameboid movement potential of macrophages. However, the intracellular content of red or orange–red lipid droplets was relatively low. Upon treatment with 100 μg/mL ox-LDL, a large number of orange and red lipid droplets were observed within the cells. Due to substantial lipid droplet accumulation, the THP-1 cells increased in volume and became rounder in morphology, presenting a distinct foam cell phenotype. Furthermore, when cells were transfected with either NC or shRNA plasmids in the presence of 100 μg/mL ox-LDL treatment, intracellular orange and red lipid droplets were also found to accumulate significantly, with no notable reduction in lipid droplet accumulation compared to the ox-LDL treatment alone group (Figure 4F). This finding suggests that inhibition of COX-2 expression may not significantly impact intracellular cholesterol accumulation, consistent with the previous results obtained from the cholesterol enzymatic assay measuring intracellular cholesterol levels.

### 2.4. Antimicrobial Peptide Chensinin-1b and Its Analogs Inhibited Wnt/β-Catenin Signaling Pathway in the ox-LDL-Induced Foaming THP-1 Cells

**Figure 5:**
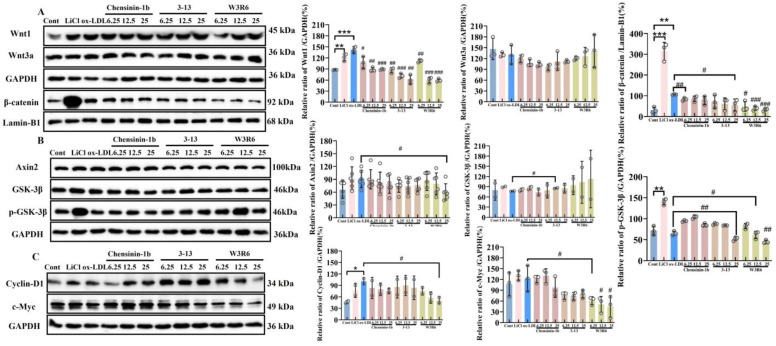
Antimicrobial peptide chensinin-1b and its analogs inhibited the Wnt/β-catenin signaling pathway in the ox-LDL-induced foaming THP-1 cells. (**A**–**C**) Western blot analysis of canonical Wnt protein expression following co-treatment with antimicrobial peptides (6.25, 12.5, 25 μM) and 100 μg/mL ox-LDL; *: Cont/ox-LDL; *: *p* < 0.05; **: *p* < 0.01; ***: *p* < 0.001; #: ox-LDL/AMPs; #: *p* < 0.05; ##: *p* < 0.01; ###: *p* < 0.001).

Previous studies have found that chensinin-1b and its analogs 3–13 and W3R6 attenuated LPS-induced inflammatory responses by inhibiting the release of inflammatory cytokines such as TNF-α and IL-6 in LPS-induced macrophage and mouse models [24,38,39]. Here, the effect of chensinin-1b and its analogs on the Wnt/β-catenin signaling pathway in the ox-LDL-induced foaming THP-1 cells. WB results showed that the addition of 100 μg/mL ox-LDL promoted the expression of the canonical Wnt ligand Wnt1 (*p* < 0.01) in THP-1 macrophages, with an increase of approximately 70% compared to the control group (Figure 5A). All three peptides demonstrated significant inhibitory effects on the expression of the Wnt1 ligand. Among the three peptides, 3–13 exhibited the most pronounced effect, reducing Wnt1 expression to approximately 40%, 50%, and 60% (*p* < 0.01). None of the three peptides had a significant effect on the expression of the Wnt3a ligand. Chensinin-1b, 3–13 and W3R6 all had a very significant inhibitory effect on the expression of β-catenin protein in the nuclei of human macrophage-like THP-1 cells induced by ox-LDL (*p* < 0.01) in a concentration-dependent manner. Among them, W3R6 exhibited the most significant inhibitory effect, with concentrations of 6.25, 12.5, and 25 μM inhibiting the expression levels of β-catenin protein in the nucleus by approximately 50%, 55%, and 70% (*p* < 0.01).

As shown in Figure 5B, LiCl did not change the expression of GSK-3β and Axin2 but significantly promoted the phosphorylation of GSK-3β at the Ser 9 site (*p* < 0.01). After co-treatment with 6.25, 12.5, and 25 μM of chensinin-1b and its analogs along with ox-LDL, it was found that treatment with higher concentrations (12.5 and 25 μM) of 3–13 and W3R6 significantly inhibited the phosphorylation of GSK-3β at the Ser 9 site (*p* < 0.05). Taking the 25 μM treatment concentration as an example, the phosphorylation level of GSK-3β was reduced to approximately 30% of that in the ox-LDL-treated group, indicating that antimicrobial peptide treatment can inhibit the classical Wnt/β-catenin signaling pathway in foam cells by enhancing the kinase activity of GSK-3β. W3R6 had a very pronounced inhibitory effect on Axin2 at 25 μM (*p* < 0.05), reducing its expression to half that of the ox-LDL group. ox-LDL increased the expression of Cyclin-D1 by 1.5-fold compared to the control group (*p* < 0.05) (Figure 5C). W3R6 effectively inhibited the expression of cyclin-D1 and c-Myc, which are downstream target genes of the canonical Wnt/β-catenin signaling pathway. Treatment with 25 μM W3R6 significantly suppressed cyclin-D1 expression, reducing it to 50% of the level observed in the ox-LDL-treated group (*p* < 0.05). Furthermore, W3R6 also demonstrated substantial inhibition of c-Myc expression in a dose-dependent manner. At concentrations of 6.25, 12.5, and 25 μM, W3R6 reduced c-Myc expression by 50%, 58%, and 62.5% (*p* < 0.05). Therefore, Chensinin-1b and its analogs not only inhibit the expression of the canonical Wnt ligand Wnt1 but also effectively suppress the nuclear translocation of β-catenin protein, without significantly affecting the expression of components of the β-catenin degradation complex. Analogs 3–13 and W3R6 demonstrated more pronounced effects compared to chensinin-1b, as they further inhibited the expression of downstream target proteins cyclin-D1 and c-Myc, thereby providing a more effective suppression of the pathway.

**Figure 6:**
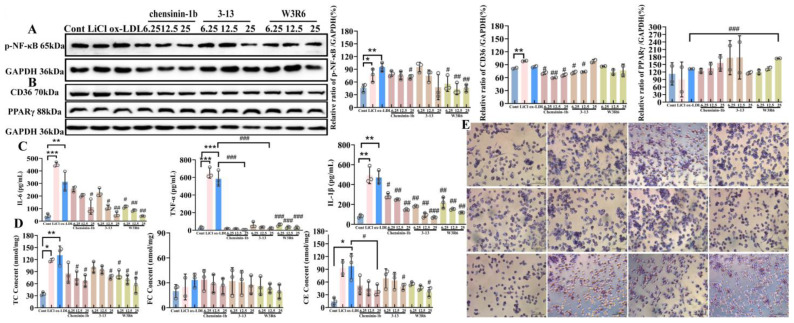
The inhibitory effects of antimicrobial peptide Chensinin-1b and its analogs on inflammation and cholesterol accumulation. (**A**) inflammatory cytokine release; (**B**) key protein expression of the NF-κB signaling pathway; (**C**) expression of lipid metabolism-related proteins; (**D**) Oil Red O staining (Scale bar = 100 µm); (**E**) TC, FC, CE; *: Cont/ox-LDL; *: *p* < 0.05; **: *p* < 0.01; ***: *p* < 0.001; #: ox-LDL/AMPs; #: *p* < 0.05; ##: *p* < 0.01; ###: *p* < 0.001).

Chensinin-1b and its analogs can exert an inhibitory effect on the inflammatory responses and the increase in intracellular cholesterol levels caused by the activation of the canonical Wnt/β-catenin signaling pathway. Elisa detection of inflammatory cytokine release in THP-1 cells revealed that treatment with both the canonical Wnt/β-catenin signaling pathway agonist LiCl and ox-LDL promoted the release of the inflammatory cytokines IL-6, TNF-α, and IL-1β. LiCl treatment increased the release of these three cytokines by approximately 10-fold, 30-fold, and 7-fold compared to the control group (*p* < 0.001), while ox-LDL promoted their release by approximately 6-fold, 30-fold, and 7-fold relative to the control group (*p* < 0.01), with the promoting effects of LiCl and ox-LDL on TNF-α and IL-1β release being comparable. Furthermore, following co-treatment with increasing concentrations (6.25, 12.5, and 25 μM) of the antimicrobial peptide chensinin-1b and its analogs 3–13 and W3R6, along with 100 μg/mL ox-LDL for 24 h, it was found that all three peptides significantly inhibited the release of the aforementioned inflammatory cytokines (*p* < 0.05). For chensinin-1b at 6.25, 12.5, and 25 μM, the inhibition rates for IL-6 release were approximately 15%, 35%, and 68%; for TNF-α release, all around 95%; and for IL-1β release, approximately 48%, 52%, and 60%. For peptide 3–13 at the same concentrations, the inhibition rates for IL-6 release were approximately 29%, 68%, and 84%; for TNF-α release, all above 90%; and for IL-1β release, approximately 52%, 76%, and 77%. For peptide W3R6 at 6.25, 12.5, and 25 μM, the inhibition rates for IL-6 release were approximately 61%, 68%, and 84%; for TNF-α release, around 83% and 90%; and for IL-1β release, approximately 48%, 57%, and 74% (Figure 6C). Collectively, these experiments indicate that the anti-inflammatory effects exerted by these antimicrobial peptides in THP-1-derived foam cells are achieved through the inhibition of the canonical Wnt/β-catenin signaling pathway activation, suggesting that this canonical pathway may interact with other pro-inflammatory signaling pathways and play a significant role in the occurrence of inflammatory responses. After examining the inhibitory effects of antimicrobial peptide treatment on the release of inflammatory cytokines in foam cells, we subsequently used Western blotting to detect changes in the phosphorylation level of the NF-κB p65, a key protein in the inflammatory signaling pathway. The results showed that ox-LDL treatment significantly promoted the phosphorylation of the p65 subunit (*p* < 0.01), with the phosphorylation level approximately doubling that of the control group. In contrast, co-treatment with W3R6 and ox-LDL reduced the phosphorylation level of p65 to approximately 30–50% of that in the ox-LDL-only treatment group, and this reduction was statistically significant (*p* < 0.01) (Figure 6A).

Treatment with 100 μg/mL of ox-LDL in PMA-induced human macrophage-like THP-1 cells for 24 h resulted in an increase in the expression of the key protein CD36, which is associated with lipid uptake, but the increase is not statistically significant (*p* > 0.05). The inhibitory effect of antimicrobial peptide chensinin-1b on CD36 expression was more significant. After co-treatment with three different concentrations of c-1b (6.25, 12.5, and 25 μM) and ox-LDL, CD36 expression was reduced by 10–30% compared to the ox-LDL alone treatment group (*p* < 0.05). The derivative peptide 3–13 also significantly inhibited CD36 expression at lower concentrations (*p* < 0.05), reducing its expression by approximately 15%. PPARγ, acting as a nuclear transcription factor for CD36, its expression was not affected by ox-LDL treatment (*p* > 0.05). However, a significant increase in the expression of PPARγ was observed after treatment with 25 μM of W3R6 (*p* < 0.001), with an increase of approximately 33% (Figure 6B).

The cholesterol levels, such as TC, FC, and CE, were also examined in PMA-induced human macrophage-like THP-1 cells. Oil Red O staining results are shown in Figure 6E. The control group cells had nuclei stained blue with almost no lipid droplets inside, while a large number of orange and red lipid droplets were observed in the ox-LDL-treated cells. The cell volume increased, and the shape became round due to the accumulation of a large number of lipid droplets. A similar result with the ox-LDL-treated cells was observed in the LiCl-treated cells: a large number of orange and red lipid droplets inside the cells. Chensinin-1b and its analogs significantly reduced lipid droplets within the cells, and the cell morphology tended to be normal. The experimental phenomena indicate that the three antimicrobial peptides can inhibit macrophage foam cell formation, and the inhibitory effect gradually increases with the concentration of the peptide used, showing a concentration-dependent relationship. The effect of chensinin-1b and its analogs on cholesterol content in human macrophage-like THP-1 cells induced by ox-LDL was detected using a cholesterol enzyme-linked assay kit. The experimental results are shown in Figure 6D: both ox-LDL and LiCl can significantly increase the total cholesterol content in cells (*p* < 0.01), approximately 2.25 times that of the control group; after treatment with the three antimicrobial peptides, the total cholesterol and cholesterol ester content in cells were reduced in a concentration-dependent manner, with W3R6 showing slightly better effects than the other two antimicrobial peptides. At 25 μM, the effects of the three antimicrobial peptides were the most significant (*p* < 0.05), with the inhibition rates of total cholesterol by chensinin-1b and its analogs being 53.8%, 42.3%, and 57.7%, but the effects of the selected three antimicrobial peptides on free cholesterol content were relatively small.

**Figure 7:**
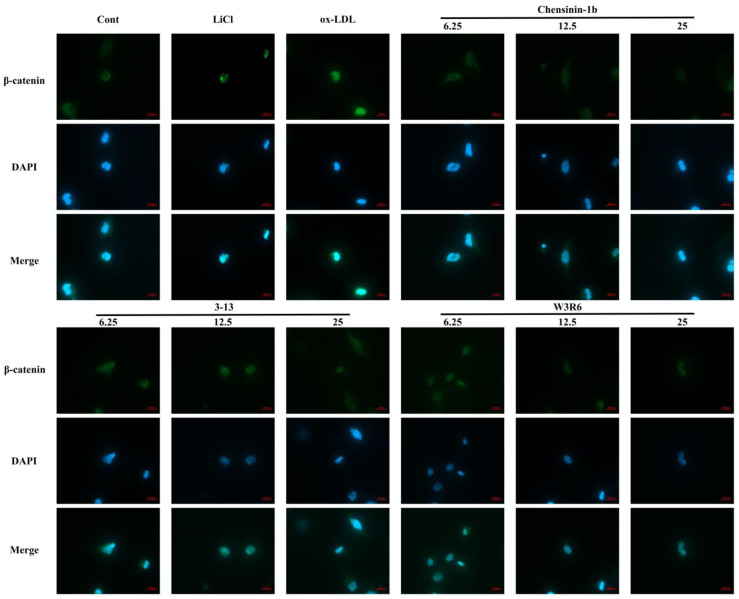
Immunofluorescence detection of the subcellular localization of β-catenin, a key protein in the canonical Wnt/β-catenin signaling pathway (stained for β-catenin (green), nuclei were counterstained with DAPI (blue). Images were acquired using a confocal microscope at 40× magnification, (Scale bar = 20 µm)).

To further evaluate the effects of the antimicrobial peptide chensinin-1b and its derived peptides on the activation of the Wnt/β-catenin signaling pathway, we employed immunofluorescence confocal microscopy to observe the subcellular localization of the key protein β-catenin (Figure 7). Immunofluorescence staining results showed that in the control group cells, β-catenin was primarily localized at the cell membrane. Treatment with 20 mM LiCl induced the appearance of strong fluorescence signals of β-catenin in the nucleus. After treatment with 100 μg/mL ox-LDL for 24 h, a significant translocation of β-catenin from the cell membrane to the nucleus was observed, characterized by intense fluorescence signals in the nuclear region, which colocalized with DAPI. In contrast, when cells were co-treated with different concentrations of antimicrobial peptides (6.25, 12.5, and 25 μM) along with ox-LDL for 24 h, the nuclear accumulation of β-catenin was markedly reduced. Under these conditions, β-catenin was primarily retained in the cytoplasm, and the fluorescence signals within the nucleus were significantly diminished. These immunofluorescence staining results indicate that the antimicrobial peptides chensinin-1b and its derived peptides 3–13 and W3R6 can downregulate the aberrant activation of the canonical Wnt/β-catenin signaling pathway induced by ox-LDL, potentially by retaining the key pathway protein β-catenin in the cytoplasm, thereby preventing its transcriptional activity in the nucleus.

## 3. Discussion

Nowadays, an increasing amount of evidence indicates that the occurrence and development of atherosclerosis are determined to be processes closely related to inflammatory responses, and inflammation plays a crucial role in plaque rupture, ultimately leading to serious complications such as myocardial infarction, causing patient death [40,41]. In AS, the activation of the canonical Wnt/β-catenin signaling pathway is associated with biological functions such as endothelial dysfunction, smooth muscle cell apoptosis, and macrophage inflammation [12,42,43,44,45,46]. This study found that ox-LDL treatment can activate the canonical Wnt/β-catenin signaling pathway in THP-1-derived foam cells by promoting the nuclear entry of the key protein β-catenin and stimulating the expression of key downstream target proteins of the pathway, including cyclin-D1, c-Myc, and COX-2. Based on the important role of the Wnt/β-catenin pathway in the progression of AS, we investigated whether the antimicrobial peptide chensinin-1b and its analogs 3–13 and W3R6 inhibit the activation of the canonical Wnt/β-catenin signaling pathway in foam cells. The results showed that treatment with chensinin-1b not only inhibited the nuclear entry of β-catenin but also significantly suppressed the expression of downstream target genes associated with the pathway, thereby downregulating the activation of the canonical Wnt/β-catenin signaling pathway in ox-LDL-induced THP-1-derived foam cells.

In the disease process of AS, multiple pro-inflammatory cytokines are abnormally expressed in macrophages [47,48,49]. This study found that ox-LDL treatment increased the release of pro-inflammatory factors such as IL-6, TNF-α, and IL-1β in THP-1 foam cells; however, the addition of the canonical Wnt/β-catenin signaling pathway inhibitor IWP-2 significantly inhibited the release of the aforementioned inflammatory factors, indicating that the inhibition of the canonical Wnt/β-catenin signaling pathway could reduce a severe inflammatory response. Furthermore, our study indicated that chensinin-1b and its analogs exerted anti-inflammatory effects by regulating the canonical Wnt/β-catenin signaling pathway, inhibiting the release of inflammatory factors and the over-activation of key proteins in the inflammatory pathway during the development process of AS, thereby inhibiting the disease process of AS.

The antimicrobial peptide chensinin-1b and its analogs 3–13 and W3R6, exert anti-atherosclerotic effects by targeting and inhibiting the activation of the canonical Wnt/β-catenin signaling pathway in foam cells, with the core molecular mechanism involving the suppression of COX-2, a key functional protein downstream of this pathway. Specifically, these antimicrobial peptides interfere with the expression or signal transduction of the Wnt1 ligand, effectively reducing the stability of β-catenin and inhibiting its nuclear translocation. This downregulates the expression of the *COX-2* gene, which is driven by the β-catenin/TCF transcription complex. The consequent reduction in COX-2 protein levels directly decreases the synthesis of its catalytic product PGE_2_. This attenuates the downstream signaling initiated by PGE_2_ binding to and activating EP2/EP4 receptors, including its facilitative effect on the activation of the IKK complex. The inhibition of IKK activity leads to reduced phosphorylation and degradation of its substrate, IκBα, allowing IκBα to more effectively retain the NF-κB p65 subunit in the cytoplasm and block its nuclear translocation. The diminished nuclear entry of p65 directly weakens the transcriptional activity of NF-κB, resulting in significant inhibition of the transcription and release of key downstream pro-inflammatory cytokines such as TNF-α, thereby functionally curtailing the local inflammatory response. In summary, these antimicrobial peptides achieve a dual inhibitory effect on both inflammation and abnormal lipid accumulation in foam cells through a complete molecular signaling cascade: inhibiting the canonical Wnt pathway, downregulating COX-2 expression, reducing PGE_2_ production, suppressing the IKK/IκB/NF-κB p65 signaling axis, and decreasing the occurrence of inflammatory responses in foam cells.

In summary, this study suggests that chensinin-1b and its analogs exert anti-atherosclerotic effects by inhibiting the Wnt/β-catenin/COX-2/NF-κB axis. Comparative analysis of the obtained data revealed distinct characteristics among the three c-1b-derived antimicrobial peptides. Previous studies from our laboratory showed that W3R6 and 3–13 exhibited lower cytotoxicity toward ox-LDL-induced foam cells than chensinin-1b; at concentrations below 25 μM, none of the three peptides significantly affected cell viability, with survival rates remaining above 75% [50]. In terms of potency, chensinin-1b showed the most pronounced inhibitory effect on TNF-α release, whereas W3R6 exhibited stronger inhibition of IL-6 and IL-1β and more effectively reduced NF-κB p65 phosphorylation. These observations may be attributed to differences in target selectivity and downstream signaling preference. Regarding intracellular cholesterol accumulation, both chensinin-1b and W3R6 showed promising inhibitory effects. Mechanistically, W3R6 was more effective in suppressing the canonical Wnt/β-catenin pathway: it not only downregulated the expression of the canonical ligand Wnt1 but also more potently inhibited β-catenin nuclear translocation and its downstream targets cyclin-D1 and c-Myc. These findings suggest that, as potential therapeutic agents for AS, these antimicrobial peptides can systematically downregulate the canonical Wnt/β-catenin pathway at three levels, namely the initiation signal, the signal amplification process involving β-catenin stabilization and nuclear entry, and the functional output of target gene expression. Based on these differences, we selected W3R6 for subsequent in vivo experiments and emphasized the importance of structural optimization in developing selective anti-atherosclerotic agents.

## 4. Materials and Methods

### 4.1. Peptide Synthesis

Frog skin peptide chensinin-1b, W3R6 and 3–13 were synthesized by GL Biochemistry Inc. (Shanghai, China). The purity of the peptide was >95%, and the amino acid sequences and the physicochemical characteristics of the peptide were summarized in Table 1.

### 4.2. Cell Culture

Human leukemia monocyte cell line THP-1 was obtained from Kaiji Biology Co. (Nanjing, China). Cells were cultured in PRMI1640 medium containing 10% fetal bovine serum and 0.05 mM β-mercaptoethanol (BME) at 37 °C and 5% CO_2_. THP-1 cells were incubated with 100 ng/mL phorbol 12-myristate 13-acetate (PMA) for 48 h to obtain the THP-1-derived macrophages. After the synchronous treatment with serum-free 1640 medium for 24 h, the THP-1-derived macrophages were incubated with 100 μg/mL of ox-LDL for another 24 h to establish the foam cell model.

### 4.3. Cytotoxicity Assay

Cytotoxicity of peptide, lithium chloride (LiCl) and IWP-2 was determined by using the CCK-8 kit. Briefly, THP-1 cells were added to 96-well plates at a density of 5 × 10^5^ cells/mL in a volume of 100 μL per well, and cultured with PMA at 37 °C, 5% CO_2_ to differentiate into macrophage phenotype. After synchronization, cells were treated with LiCl (0, 1, 5, 10, 20, and 40 mM) or IWP-2 (0, 1, 5, 10, and 20 μM) for 24 h, respectively. An amount of 10 μL of CCK-8 reagent was added, and the cells were incubated for 2 h under dark conditions. The absorbance value at 450 nm was measured at 490 nm with a Multiskan FC microplate reader (Thermo Fisher Scientific, Waltham, MA, USA).

### 4.4. Pro-Inflammatory Factors Assays

Pro-inflammatory factors IL-6, IL-1β, and TNF-α were detected by using an enzyme-linked immunosorbent assay (ELISA) kit according to the manufacturer’s instructions (Neobioscience, Shenzhen, China). THP-1 macrophages were treated with ox-LDL, or LiCl, or IWP-2 for 24 h, respectively. And then the release of pro-inflammatory cytokines IL-6, IL-1β, and TNF-α in the supernatants was measured at 450 nm by Multiskan FC microplate reader (Thermo Fisher Scientific, USA).

### 4.5. Cholesterol Detection

THP-1 macrophages were treated with ox-LDL and/or chensinin-1b and its analogs/ or LiCl/ or IWP-2 in each group for 24 h. After washing in PBS three times, cells were lysed with 100 μL of lysis buffer. Total cholesterol (TC) and free cholesterol (FC) in the collected supernatant were detected by the total cholesterol and free cholesterol assay kits according to the manufacturer’s instructions (Solarbio, Shanghai, China). Cholesteryl ester (CE) was calculated using the following formula: CE = TC − FC.

Oil red O staining THP-1 macrophages were treated with ox-LDL and/or chensinin-1b and its analogs/ or LiCl/ or IWP-2 for 24 h. Lipid droplets were observed by Oil Red O (ORO) staining (Solarbio, Shanghai, China) for 15 min at room temperature. Images were observed using a light microscope (Leica, Germany).

### 4.6. Quantitative Real-Time PCR

THP-1 macrophages were cultured with ox-LDL and/or chensinin-1b and its analogs at 37 °C and 5% CO_2_ for 24 h. An amount of 1 mL of total RNA Trizol reagent (Invitrogen, Shanghai, China) was added to each well to extract the total RNA. Reverse transcription PCR reaction was performed using a Super Script™ III kit (Invitrogen, Shanghai, China), and cDNA was amplified in an ABI-Prism 7500 Fast sequence detection system (Applied Biosystems, Waltham, MA, USA). After a normalization of GAPDH, the relative mRNA expression levels were calculated by using the 2^−∆∆CT^ method according to the calculation formula: ΔCt = Ct_target gene_ − Ct _GAPDH_; ΔΔCt = ΔCt − ΔCt_control_; *n* = 2^−ΔΔCt^. The primer sequences were summarized in Table 2.

### 4.7. Western Blot

THP-1 macrophages were cultured with ox-LDL and/or chensinin-1b and its analogs at 37 °C and 5% CO_2_ for 24 h. After washing in PBS three times, cells were lysed with RIPA lysis buffer. The protein concentration was detected using the BCA Protein Assay Kit according to the manufacturer’s instructions (Meilunbio, Dalian, China). And then proteins were separated by 10% sodium dodecyl sulfate-polyacrylamide gel electrophoresis (SDS-PAGE) and electro-transferred to a polyvinylidene fluoride (PVDF) membrane. After blocking with 5% skim milk in TBST at room temperature for 2 h, the membranes were incubated with primary antibody overnight at 4 °C against the antibodies (anti-β-catenin antibody: #8480, 1/1000; anti-GSK-3β antibody: #12456, 1/1000; anti-phosphoGSK-3β antibody: #5558, 1/1000; anti-PPARγ antibodies: #2435, 1/1000; anti-Wnt1 antibody: ER65317, 1/1000; anti-Wnt3a antibodies: HA500193, 1/1000; anti-cyclin-D1 antibody: WL01435a, 1/1000; anti-c-Myc antibody: WL01787, 1/1000; anti-phospho NF-κB antibodies: WL02169, 1/1000; anti-Axin2 antibody: A2513, 1/1000; anti-GAPDH antibody: A19056, 1/5000; anti-Lamin B1 antibody: A1910, 1/1000; abclonal). Then the membrane was incubated with secondary antibody (HRP-conjugated goat anti-rabbit IgG: AS014, 1/5000, abclonal) for 1 h at room temperature and developed with ECL reagent (Tanon Science & Technology Co., Shanghai, China), and visualized by an Azure Biosystems c500 instrument. Lamin B1 and GAPDH were used for normalization of the relative protein expression levels in nuclear protein and total protein, respectively. Protein quantification was performed using ImageJ software (version 1.53e, National Institutes of Health, Bethesda, MD, USA).

### 4.8. Cell Transfection

THP-1 cells were seeded into 6-well plates at a density of 3 × 10^5^ cells/mL and treated with 100 ng/mL PMA for 48 h to induce adherence. According to the instructions for the Zeta Life Advanced DNA RNA Transfection Reagent, the plasmid and transfection reagent were diluted at a 1:1 ratio in Opti-MEM reduced serum medium and incubated at room temperature (RT) for 15 min (plasmid sequences are provided in Table 2). The mixed transfection solution was then added dropwise to the cell culture plate. The plate was gently swirled to ensure even distribution of the transfection mixture and returned to the incubator for 24 h. Subsequently, the cells were treated with 100 μg/mL ox-LDL for an additional 24 h for subsequent experiments.

### 4.9. Immunofluorescence

THP-1 cells were seeded into a 12-well plate at a density of 5 × 10^4^ cells/mL, with coverslips placed in the wells to allow cell growth on the coverslips. Each well was supplemented with 500 μL of the cell suspension. After induction of differentiation and drug treatment for 24 h in each group, the cells were washed three times with PBS, fixed with paraformaldehyde for 25 min at room temperature, and then washed three times with PBS again. Permeabilization was performed with 0.1% Triton X-100 for 15 min at room temperature, followed by three washes with PBS. The cells were blocked with 0.5% BSA prepared in PBS for one hour, after which the primary antibody (anti-β-catenin antibody: E247, 1/250, Abcam, Cambridge, UK) was added directly and incubated overnight at 4 °C. After washing three times with PBS on a shaker at room temperature (5 min per wash), the secondary antibody, diluted at 1:100, was added and incubated in a constant temperature incubator at 37 °C for 1 h. The cells were then washed three times with PBS on a shaker at room temperature (5 min per wash). Subsequently, 500 μL of Hoechst 33342 was added, and the cells were incubated in a constant temperature incubator at 37 °C for 25 min. After three washes with PBS, an antifade mounting medium was applied, and the samples were observed and photographed under a confocal microscope.

### 4.10. Statistical Analysis

Data from three independent experiments were analyzed using SPSS statistical software (version 26.0) and expressed as mean ± standard deviation (SD). Student’s *t*-test was used for comparison between the two groups. One-way analysis of variance (ANOVA) was used for comparison between groups, and the difference was statistically significant with *p* < 0.05.

## 5. Conclusions

This study is the first to reveal that antimicrobial peptide chensinin-1b and its analogs can alleviate inflammation and increased cholesterol levels induced by ox-LDL by regulating the canonical Wnt/β-catenin signaling pathway, and it demonstrates the potential of chensinin-1b and its analogs in the treatment of AS.

## Figures and Tables

**Table 1:** Physicochemical information of AMPs.

Frog Skin Peptide	Amino Acid Sequences	Molecular Weight	Hydrophobicity Value	Charge Number
chensinin-1b	SKVWRHWRRFWHRAHRKL	2542.9	9.69	7
W3R6	VWRRWRRFWRR	1760.1	13.03	6
3–13	VWRHWRRFWHR	1722.1	12.92	4

**Table 2:** COX-2 shRNA plasmid sequence.

Sh-RNA Plasmid	Sequences
PTGS2-Human-1	5′-GCTGAATTTAACACCCTCTAT-3′
PTGS2-Human-2	5′-CCATTCTCCTTGAAAGGACTT-3′
PTGS2-Human-3	5′-GCAGATGAAATACCAGTCTTT-3′
NC	5′-GUACUUUUGUGUAGUACAAUU-3′

## Data Availability

The original contributions presented in this study are included in the article. Further inquiries can be directed to the corresponding author.
